# Successful retrieval of tip-embedded inferior vena cava filter using a modified forceps technique: case report

**DOI:** 10.1186/s12959-024-00595-7

**Published:** 2024-03-12

**Authors:** Yang Liu, Junlong Ma, Qiqi Wang, Wei Zeng, Chunshui He

**Affiliations:** 1 Department of Vascular Surgery, Hospital of Chengdu University of Traditional Chinese Medicine, 610072 Chengdu, Sichuan, CN China; 2 Department of Hepato-biliary-pancreatic and Vascular surgery, Meishan municipal people’s hospital, Meishan, Sichuan, CN China

**Keywords:** IVC filter, Tip-embedded, Retrieval

## Abstract

**Background:**

The retrieval of inferior vena cava (IVC) filter is essential for preventing complications associated with the device. Advanced techniques have been developed to improve the success rate of retrieving tip-embedded filters. The forceps technique is frequently used to address this issue.

**Case presentation:**

We present a case study of two patients who underwent a successful tip-embedded IVC filter retrieval using a modified forceps technique, which has not been previously reported. This technique involves using a wire loop under the filter tip and a forceps to grasp the filter shoulder. By pulling the wire loop and pushing the forceps in counterforce, the filter tip is straightened and aligned with the vascular sheath. The vascular sheath can then dissect the filter tip out from the caval wall and get inside the sheath to complete the retrieval.

**Conclusions:**

The modified forceps technique we present here offers a new solution for the complex retrieval of IVC filters.

## Background

Retrievable inferior vena cava (IVC) filters are employed to prevent pulmonary embolism (PE) in patients with lower-extremity deep vein thrombosis (DVT) when anticoagulation is contraindicated. IVC filter retrieval is advised after the resolution of PE risk [1–3]. Prompt retrieval of IVC filters is essential for preventing complications associated with the device, including filter fracture, vena cava perforation, filter migration, filter fragment embolization, and IVC thrombosis, in order to ensure patient safety [2, 4]. The 2020 multi- society consensus statement, which involves the Society of Interventional Radiology, the ACC, and the Society for Vascular Surgery, advises the removal of IVC filters if the benefits outweigh the clinical risk [5].

Several advanced techniques have been developed to improve the effectiveness of IVC filter retrieval when the standard snare technique is not possible or fails [6]. These techniques include the utilization of additional snares, the wire-loop snare technique, angioplasty balloons, the forceps technique (endobronchial or endoscopy forceps), as well as the application of the excimer laser sheath [7]. Notably, the forceps technique has been extensively documented in large-scale studies, demonstrating high success rates and minimal complications [8, 9].

In this study, we initially presented a modified forceps technique that addresses the shortcomings of the conventional forceps method. Using this approach, we successfully extracted the tip-embedded IVC filters in two instances, without encountering any complications.

## Case presentation

Case 1 was a 33-year-old male patient who experienced trauma developed DVT in the right femoral vein, iliac vein, and IVC. In order to prevent a potentially fatal PE, the patient underwent the implantation of a retrievable IVC filter (Denali, Bard PV, Tempe, USA). The patient received standard anticoagulation therapy for 6 months. However, despite using the forceps technique at another hospital, the retrieval of the filter was unsuccessful due to thrombosis in the filter and the tip being embedded. Subsequently, he was referred to our department for the extraction of the filter.

**Fig. 1:**
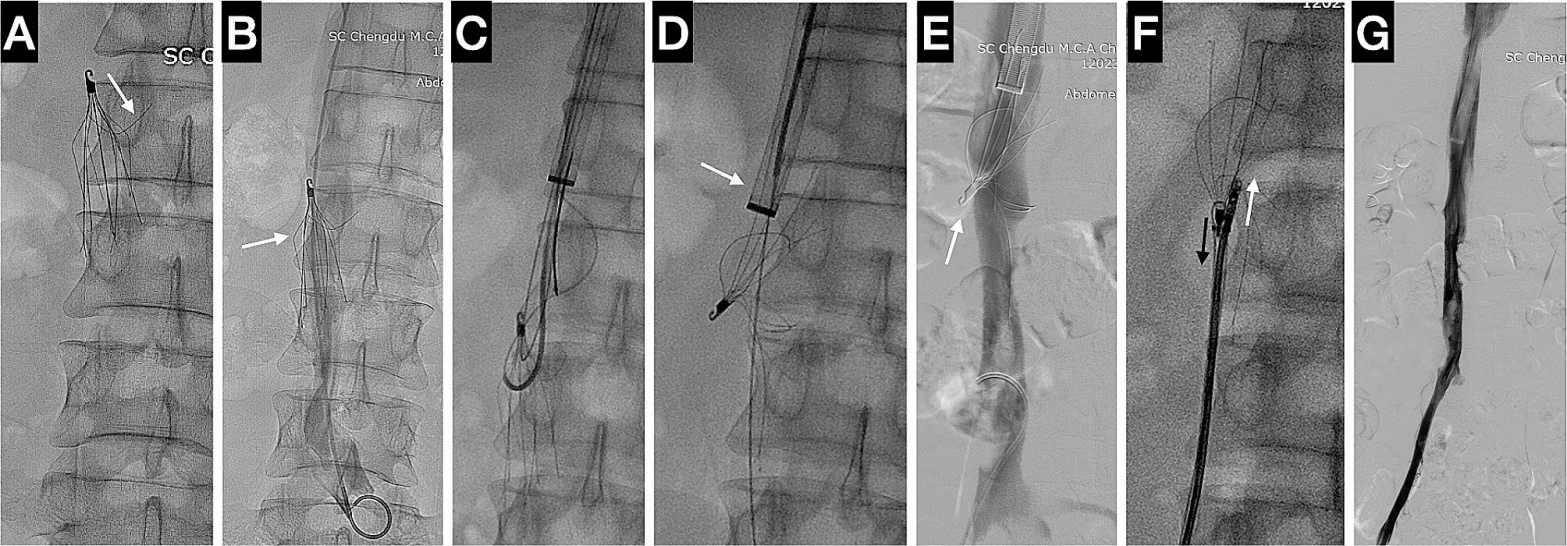
The filter retrieval in case 1. **(A)** The initial inspection of the filter demonstrated that one strut had been inverted due to the previous attempt to retrieve the filter (arrow). **(B)** Multiplanar venography confirmed filter embedding of the tip and upper side (arrow). **(C)** A pigtail catheter was used to hook under the apex of the filter, while a 0.035-inch, 260 cm length guide wire was passed through the catheter and retrieved using a snare from the sheath. **(D)** The filter was inverted, and certain struts were displaced within the sheath (arrow). **(E)** The venography conducted from the femoral vein revealed that the tip was still embedded. **(F)** By applying a pulling force from the wire loop (black arrow) and a pushing force from the forceps (white arrow) at the same time, the filter’s tip was straightened and aligned with the sheath. **(G)** The final venography showed no perforation in the IVC, but there was still residual thrombosis in the right iliac vein and IVC

Given the prolonged dwell time (over 7 months), the occurrence of thrombosis in the filter, the embedded tip, and the unsuccessful attempts using forceps techniques, it was decided to employ the forceps technique in conjunction with a large sheath from the outset of the treatment, as recommended by Tavri et al. [9]. The procedure was performed under local anesthesia. A 20 F vascular sheath (DrySeal, Gore Medical, Newark, USA) was positioned just above the filter, accessed through the right internal jugular vein. The initial image of the filter revealed that one strut had been inverted (Fig. 1A, arrow) as a result of the previous filter retrieval attempt. Multiplanar venography was performed, which confirmed that the tip and a portion of the upper side of the filter were embedded (Fig. 1B, arrow). Initially, the wire loop was created to assist the flexible endoscopy forceps in capturing the filter tip because the flexible forceps lacked direction. A pigtail catheter (Terumo, Tokyo, Japan) was used to hook under the apex of the filter, while a 0.035-inch, 260 cm length guide wire (Terumo, Tokyo, Japan) was passed through the catheter and subsequently captured using a snare (Bard PV, Tempe, USA), and the end was extracted from the 20Fr sheath (Fig. 1C). Traction was applied to both ends of the guide wire, enabling the gradual downward movement of the sheath towards the filter tip. A flexible endoscopy forceps (FB-A-1, Kangjin Medical, Changzhou, China) was then inserted into the sheath, attempting to grasp the section of the filter closest to the tip. In order to oversheath the filter tip, employ a forward counterforce by pushing the sheath while vigorously retracting the forceps. Despite the intense force causing some twisting and shrinkage of a short segment of the sheath outside the body, the efforts proved futile as the tip’s apex failed to align with the sheath. The endeavor led to the inversion of the filter and displacement of certain struts within the sheath (Fig. 1D, arrow).

Then, access to the right femoral vein was secured in order to perform the downward retrieval of the filter. An 11 F vascular sheath (Bard PV, Tempe, USA) was positioned just below the filter. The venography revealed that the tip remained implanted (Fig. 1E, arrow). The wire loop was once again employed, utilizing a new pair of forceps to carefully grasp the upper section of the filter. By simultaneously applying a pulling force from the wire (Fig. 1F, black arrow) and a pushing force from the forceps (Fig. 1F, white arrow), the tip of the filter was straightened and aligned with the sheath. The assistant then advanced the sheath, dislodging the filter from the caval wall and guiding it into the sheath. After verifying the presence of the filter tip within the sheath through multiple projections, the forceps were opened and retracted from the body. The filter was effectively retrieved by employing loop wire traction and sheath counterforce. The final venography revealed no perforation in the IVC, although residual thrombosis was still present in the right iliac vein and IVC (Fig. 1G).

**Fig. 2:**
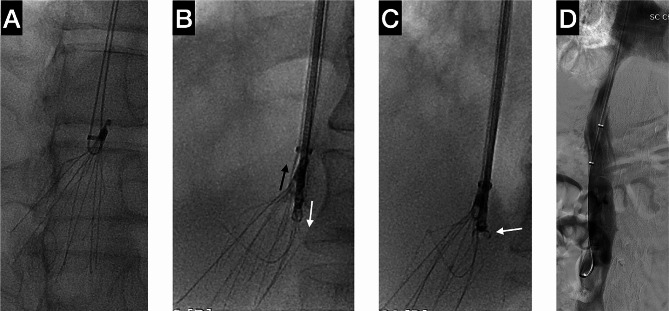
The filter retrieval in case 2. **(A)** The wire loop was created initially. **(B)** The wire loop (black arrow) was pulled, while the forceps (white arrow) were pushed at the same time, in order to align the tip of the filter with the sheath. **(C)** After confirming that the tip was located within the sheath, the forceps were opened and drawn back (arrow), and the wire was firmly pulled to retrieve the filter. **(D)** The final venography of the IVC did not reveal any perforation

Case 2 involved a 58-year-old male patient who experienced left lower limb DVT and PE. He underwent endovascular mechanical thrombectomy for the DVT and PE. During the procedure, a Denali filter (Bard PV, Tempe, USA) was implanted. The patient was placed on a standard anticoagulation therapy regimen and a filter retrieval procedure was scheduled for two months later. The procedure was performed under local anesthesia. An 11 F vascular sheath (Bard PV, Tempe, USA) was inserted above the filter via the right internal jugular vein. After confirming the tip-embedding, the same modified forceps technique was applied using the same devices. The wire loop was created by placing a pigtail catheter (Terumo, Tokyo, Japan) under the filter’s apex, then passing a 0.035-inch, 260 cm guide wire (Terumo, Tokyo, Japan) through the catheter and capturing it with a snare (Bard PV, Tempe, USA). The wire end was extracted from the 11Fr sheath. Utilizing the identical forceps (FB-A-1, Kangjin Medical, Changzhou, China), the upper portion of the filter was grasped by slightly tightening the wire loop guide. Subsequently, by applying a pulling force from the wire loop and a pushing force from the forceps, the tip of the filter was straightened and aligned with the sheath. The sheath was then used to dissect the tissue around the tip. Once the tip was confirmed to be inside the sheath, the forceps were opened and retracted (Fig. 2C, arrow), and the wire was pulled firmly to retrieve the filter. The final venography revealed no perforation in the IVC (Fig. 2D).

## Discussion

This study presented a modified forceps technique that, to the best of our knowledge, has not been previously reported. Unlike the forceps techniques developed earlier, which often involve using forceps for dissection and retraction of the filter, this innovative method simultaneously applies both pulling (via a wire loop) and pushing (via forceps) forces to the upper part of the filter. This generates a force line that not only straightens the filter but also aligns it with the sheath. This is because all three forces - the two from the technique and the push force from the sheath - are aligned on the same line. The results demonstrated the effectiveness and safety of this technique, establishing it as a feasible innovative approach for handling complicated IVC filter retrievals involving realignment and dissection.

There are several factors associated with the failure of standard retrieval techniques. These include the presence of an embedded and tilted filter tip, a prolonged dwell time, and thrombosis within the filter [10, 11]. To enhance the success rate of IVC filter retrieval, advanced techniques have been developed. These advanced techniques are defined as methods that go beyond the simple use of a snare and sheath. Al-Hakim et al. conducted a comparative analysis of advanced filter retrieval techniques and standard techniques [12]. The results revealed that advanced techniques had a significantly higher success rate of filter retrievals (94.7%) compared to standard techniques (73.2%). Desai et al. conducted a retrospective study on 762 retrieval procedures. Their findings indicated that when standard retrieval techniques were unsuccessful, advanced techniques were deemed necessary and employed 18% of the time [10].

One of the most common challenges in filter retrieval is dealing with an IVC filter that has become embedded in the apex with fibrosis present [11]. While there are some advanced techniques to address this issue, the forceps technique was proved the most efficient technique [8, 9, 13]. In a study conducted by Zhong et al., endobronchial forceps were initially employed to extract embedded IVC filters from a total of 535 patients. The researchers reported a success rate of 98.7% in their retrieval attempts, demonstrating that this approach can effectively circumvent the need for unnecessary snare removal attempts, thereby reducing fluoroscopy usage, procedure duration, and associated costs [8]. Tavri et al. conducted a study where endobronchial forceps were used to retrieve IVC filters from 60 patients when standard retrieval techniques were unsuccessful. They achieved success in 58 cases (96.7%) but also encountered complications in 4 cases [9]. The study suggested that factors such as filter tilt, caval penetration, and filter fracture could indicate the necessity of using forceps as the primary retrieval technique.

The forceps technique, which has been described in prior literature, can be performed using either rigid endobronchial forceps or flexible endoscopy forceps [13, 14]. Typically, these forceps are employed for the dissection of neointimal and fibrotic tissue surrounding the filter tip, as well as for the subsequent retrieval of the filter. The forceps are positioned close to the filter tip under spot magnification fluoroscopy. They are then used to gently dissect the IVC filter tip from the IVC wall by removing the tissue around the filter tip. After confirming contact between the forceps and the metal hook or tip of the IVC filter, the forceps are used to grasp the tip. Subsequently, a large sheath was employed to encase the filter tip. This sheath was then utilized to dissect and remove the legs from the wall of the IVC.

The primary complication associated with the forceps technique is the formation of an IVC pseudoaneurysm [15]. This can occur if the operator unintentionally grasps the caval wall. However, the incidence of this complication is relatively low [12]. Another limitation of the forceps technique is that its success is highly dependent on the type of forceps used. Some authors [9], have emphasized the importance of selecting the appropriate forceps for use, taking into account parameters such as length, neck angle, and jaw type. To date, no studies have definitively demonstrated which type of forceps is most effective for filter retrieval. Furthermore, the selection is often limited by the hospital’s routine inventory. In certain situations, such as in our first case, the neointimal and fibrotic tissue around the filter tip can become excessively large due to prolonged dwell time and severe filter tilt. In these instances, the jaws of the forceps may be too small to grasp and remove the tissue.

**Fig. 3:**
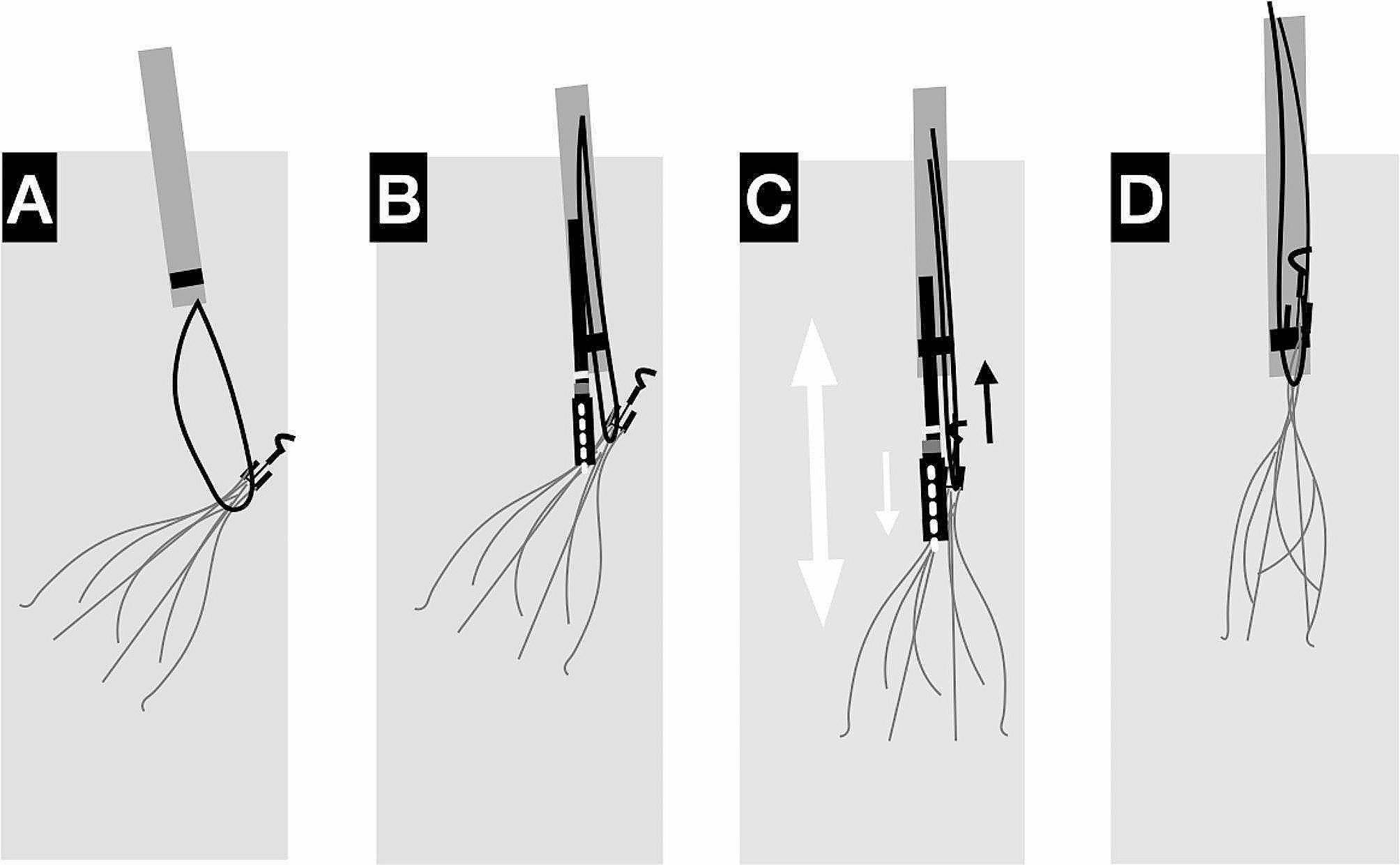
The illustration of the modified forceps technique. **(A)** The wire loop was initially performed under the tip of the filter. **(B)** The forceps, along with the wire loop, were utilized to grasp the upper portion or shoulder of the filter. **(C)** The operator exerted a pulling force on the wire loop (black arrow) and a pushing force on the forceps (white arrow). This action generated a line of force that straightened the filter (double arrow). **(D)** Complete the filter retrieval by pulling the wire loop and advancing the sheath forward

A few studies [9, 16, 17] have described the application of both forceps and wire-loop snare techniques. In cases where the wire-loop snare technique was unsuccessful as the initial attempt, the forceps technique was applied as a backup. However, our modified forceps technique is distinctly different from these methods. The wire loop was initially employed to aid the forceps in grasping the upper portion of the filter (Fig. 3A). The forceps, facilitated by the wire loop, were used to easily grasp the upper part or shoulder of the filter (Fig. 3B). The assistant partially retracted the vascular sheath, while the operator applied a pulling force through the wire loop and a pushing force through the forceps. This process created a line of force that straightened the filter, thereby generating a ‘sling’ effect to extract the tip from the fibrosis (Fig. 3C). Furthermore, it facilitated the alignment of the tip with the sheath. By advancing the vascular sheath, the assistant achieved alignment of the filter tip with the sheath, capitalizing on the alignment of all three forces along the same line. The sheath then dissected the fibrotic tissue, ultimately facilitating the insertion of the filter tip into the sheath(Fig. 3D). Once the position of the filter tip inside the sheath was confirmed, the forceps were opened and withdrawn from the sheath. By solely pulling the wire loop and pushing the sheath forward, a counterforce is created to accommodate the excess neointimal hyperplasia and fibrosis around the tip or strut, thereby completing the retrieval process.

This modified forceps technique offers several advantages. Firstly, it eliminates the need for the forceps to grasp and dissect the fibrotic tissue around the filter tip, thereby eliminating the risk of perforating the IVC wall. Secondly, this technique has a low threshold and is actually quite simple to use when grasping the upper part or the shoulders of the filter. Compared to grasping the tip of the filter, this method is significantly easier and less dependent on the instrument.

The potential risk of this technique is the fracture of the IVC filter when forceps are used to grasp the shoulder of the filter and apply a pushing force, similar to what may occur with the conventional forceps technique [13]. Posham et al. applied the filter eversion technique by clamping over the neck of the filter in 25 cases [18], whereas Matsumoto et al. utilized the grasp-and-fold technique by gripping at the midportion of the filter in 14 cases [19] when attempts to dissect the filter tip from the IVC wall using forceps were unsuccessful. They employed a large sheath to exert a counterforce to the forceps retraction in order to either evert or fold the filter inside the sheath. Despite not encountering complications such as IVC filter fracture in their reporting, the potential for IVC filter issues remains present, even though the force applied to the filter by forceps in our technique is considerably lower compared to other techniques.

## Conclusion

This study presents a modified forceps technique, previously unreported, for the retrieval of complicated IVC filters. The successful retrieval of tip-embedded inferior vena cava filters in two cases demonstrates the efficacy and safety of this modified technique. This technique enriches the repertoire of advanced filter retrieval options. Further studies employing this modified forceps technique are warranted to substantiate its benefits.

## Data Availability

No datasets were generated or analysed during the current study.

## Abbreviations

IVC: Inferior vena cava
PE: Pulmonary embolism
DVT: Deep vein thrombosis
